# Growth differentiation factor-15 levels and the risk of contrast induced nephropathy in patients with acute myocardial infarction undergoing percutaneous coronary intervention: A retrospective observation study

**DOI:** 10.1371/journal.pone.0197609

**Published:** 2018-05-23

**Authors:** Ling Sun, Xuejun Zhou, Jianguang Jiang, Xuan Zang, Xin Chen, Haiyan Li, Haitao Cao, Qingjie Wang

**Affiliations:** Department of Cardiology, Changzhou No.2 people’s Hospital, Affiliated to Nanjing Medical University, Changzhou, China; Kaohsiung Medical University Hospital, TAIWAN

## Abstract

**Aims:**

To investigate the association between growth differentiation factor-15 (GDF-15) and contrast-induced nephropathy (CIN) in patients with acute myocardial infarction (AMI) undergoing percutaneous coronary intervention (PCI).

**Methods:**

A total of 311 patients with AMI were studied retrospectively. All patients were divided into two groups according to the occurrence of CIN after PCI. Baseline clinical data were compared between two groups. Multivariate logistic regression analysis was used to identify the risk factors for CIN. Cox regression analysis was used to identify the association between GDF-15, CIN and short-term outcome.

**Results:**

There were 80 patients in CIN group (average age was 71.60 ± 13.00 years; 67.5% male) and 231 patients in non-CIN group (average age was 63.80 ± 11.70 years; 71.9%male). The concentration of GDF-15 in CIN group was higher than that of non-CIN group (1232 ± 366.6 ng/L vs. 939.20 ± 309.6 ng/L, P <0.001). According to GDF-15 quartiles, patients were divided into four groups. Multivariate logistic model indicated that the highest quartile(Q4) was significantly associated with an increased risk of CIN compared with lower level of GDF-15 (Q1, Q2 and Q3) (OR : 3.572, 1.803–7.078, P < 0.001). Of 243 patients who could calculate the ACEF risk score, area under the curve (AUC) of GDF-15 was 0.793, 95%CI: 0.729–0.856, P < 0.001, while AUC of ACEF was 0.708, 95%CI: 0.630–0.786, P < 0.001. Using 10% and 30% as arbitrary thresholds to define patients at low, intermediate, and high risk, GDF-15 achieved a net reclassification improvement (NRI) of 0.32 (95%CI: 0.123–0.518, P = 0.001) compared with the ACEF risk score. Cox regression model showed that high concentration of GDF-15 (Q4) was significantly associated with an increased risk of all-cause mortality and major adverse clinical events (MACE) (HR: 8.434, 95%CI: 2.650–26.837, P <0.001; HR: 3.562, 95%CI: 1.658–7.652, P = 0.001) compared with low level of GDF-15 (Q1, Q2 and Q3). CIN was an independent predictor of all-cause mortality and MACE in AMI patients (HR: 3.535, 95%CI: 1.135–11.005, P = 0.029; HR: 5.154, 95%CI: 2.228–11.925, P <0.001).

**Conclusion:**

GDF-15 levels increased in CIN group in AMI patients underwent PCI. GDF-15 was an independent risk factor for CIN in AMI patients underwent PCI. GDF-15 level and CIN are independent risk factors for all-cause mortality and MACE in short-term follow-ups.

## Introduction

The prognosis of patients with acute myocardial infarction (AMI) was improved by percutaneous coronary intervention (PCI). However, this radiotherapy process requires contrast medium (CM), which is becoming an important source of iatrogenic disease called contrast-induced nephropathy (CIN)[1]. CIN is the leading cause of acute kidney injury, leading to a short-term and long-term cardiovascular and renal morbidity and mortality[2]. The pathogenesis of CIN is uncertain. Possible pathophysiological mechanisms may be related to endothelial dysfunction, oxidative stress and distribution of renal blood flow[3].

In order to prevent CIN, it is necessary to identify patients at high-risk before procedure. There are several risk factors for CIN, including baseline renal function, age, gender, diabetes mellitus, heart failure, and the volume of contrast medium[4, 5]. Risk scores are useful in predicting CIN after PCI in Patients with AMI. However, all risk scores exhibited low predictive accuracy of CIN and 3-year MACEs[6]. Thus, it is necessary to explore an early predictive biomarker of CIN.

Growth differentiation factor (GDF-15) is a transforming growth factor-β cytokine which weakly expressed in normal tissues. In response to oxidative stress, endothelial dysfunction, inflammation and tissue injury, GDF-15 was increased expressed[7]. It is reported that GDF-15 are associated with increased risks for patients with acute myocardial infarction[8]. In this study, we aim to explore the association between GDF-15 and CIN in AMI patients after PCI.

## Methods

### Ethical considerations

This research was approved by Changzhou No.2 People’s Hospital ethics committee in February 2017 and this study was carried out to the Declaration of Helsinki. Prior to the use of their medical records in the study, all patients (except for death) signed written informed consent. All AMI patients in our study were enrolled into the single disease (AMI) clinical pathways system in our hospital. Patients or their trustee signed the informed consent of “single disease (AMI) clinic pathways” which stated the collection and use of blood samples for future research.

### Study populations

This study was conducted at Changzhou No.2 People’s Hospital in Jiangsu, China. Patients were recruited to the study from March 2017 after we obtained written informed consent and ethics approval for this study. Medical records of inpatients with AMI from March 2013 to October 2016 were reviewed retrospectively in department of cardiology. All eligible patients were more than twenty years old and were diagnosed with AMI. The definition of AMI was according to “the third universal definition of myocardial infarction from the Joint ESC/ACCF/AHA/WHF Task Force” [9]. All enrolled patients received PCI therapy after admission. The exclusion criteria were pregnancy, inflammatory, end stage renal disease (estimated glomerular filtration rate, eGFR, < 15 ml/min/1.73 m^2^), trauma, surgery, autoimmune disease, malignant tumor, malignant anemia, severe hepatic dysfunction, old myocardial infarction, valvular heart disease, myocarditis, pericarditis, severe sepsis, and previous heart surgery. Among 374 patients enrolled, 63 patients were excluded (31 patients refused to sign the written inform consent; 18 patients could not be contacted; and 14 patients were basic data incompleteness).

### Data collection

The following variables were collected from patients’ medical records: age, gender, heart rate, systolic blood pressure (SBP), diastolic blood pressure (DBP), usual cardiovascular risk factors (smoking, drinking, hypertension, and diabetes), and regular medications. All patients received PCI therapy. Procedural characteristics were recorded including contrast volume, contrast exposure time, number of stents, the use of contrast agent, and whether received hydration treatment or primary PCI.

### Laboratory parameters and biomarkers test

Serum samples were acquired by venipuncture on patients’ admission and were stored at -80°C. The measurements included: white blood cell count (WBC), creatinine (CR), total cholesterol (TC), low-density lipoprotein cholesterol (LDL-C), high-density lipoprotein cholesterol (HDL-C), uric acid, albumin, heamoglobinA1c (HbA1c), hemoglobin, thyrotropin stimulating hormone (TSH), and GDF-15. All biochemical analyses were conducted by inspectors blinded to the clinical data of the patients following the standard techniques.

### GDF-15 test

3 mL blood was collected into an EDTA anticoagulant tube, incubated at room temperature for 2 h, and centrifuged. GDF-15 was measured using enzyme-linked immunosorbent assay (ELISA). The detection limitation was 4.39 pg/mL and a linear range from 23.4–1500 pg/mL (Quantikine, R&D Systems, USA). The color intensity was measured at 450 nm using spectrophotometer (BioTek, Winooski, VT, United States).

### Definition of CIN, eGFR, and ACEF risk score

Serum creatinine was tested on admission or within 72 h after PCI (on admission and within 72 h for emergency PCI, or 1 h before and within 72 h for selective PCI). CIN was defined as “an absolute increase of ≥ 0.5mg/dL or a relative increase of ≥25% from baseline creatinine concentrations within 72 hours of contrast exposure”[10]. eGFR was calculated using “Modification of Diet in Renal Disease (MDRD) formula according to the serum creatinine concentration”[11]. ACEF risk score was calculated according to Ranucci et al, using the following formula: age/ejection fraction(%) + 1 (if serum creatinine ≥2.0 mg/dL) [12].

### Outcome measurements

Our primary outcome was the incidence of CIN after PCI. The secondary outcomes of the study were 30-days all-cause mortality and major adverse clinical events (MACE). MACE included death, target vessel revascularization and nonfatal myocardial infarction. 30 days all-cause mortality or MACE events were recorded by trained nurses or doctors by telephone contacts.

### Statistical analysis

Analyses were performed using R version 3.4.1 (the R Core Team; 2017 R; a programming environment for data analysis and graphic) and SPSS software (version 22.0, IBM Corp. Armonk, NY, USA). Missing data other than serum creatinine concentration or GDF-15 was present in less than 2% of the record.

The sample size was verified according to the rule that outcome events number must be 10 of each independent predictor. In our research, the sample size was no less than 300, to accommodate no more than 6 predictors in a multivariable logistic regression analysis under the assumption of at least 20% incidence of CIN[13].

Mean ± standard deviation or median and 25 and 75 percentiles were used to represent continuous variables. The categorical variable is represented by absolute value (percent). Student t test or Wilcoxon rank sum test were used to compare the differences between the two groups for continuous variables and χ2 or Fisher exact test for categorical variables. Randomized analysis of variance (ANOVA) was used to compare the continuous variables in multiple groups. Restricted cubic spline curve was used to display the association of GDF-15 and incidence of CIN. Multivariate logistic regression analysis was used to determine whether GDF-15 was an independent predictor of CIN. The adjusted odds ratio (OR) and 95% confidence interval (CI) were also calculated. The prediction value of GDF-15 and other biomarkers was determined by receiver operating characteristics (ROC) analysis. Area under the receiver operator characteristic (AUROC) curve of predictors were measured. ROC analysis was also used to compare GDF-15 with the ACEF risk score. To compare the predictive value of GDF-15 and ACEF risk score, net reclassification improvement (NRI) was also calculated by R software. The associations between GDF-15 and short-term results were compared with Kaplan-Meier survival analysis and log rank test. Cox proportional risk was used to evaluate the associations between GDF-15 and short-term results. C-statistics were calculated to evaluate if there were incremental trends in C-statistics when GDF-15 was included in the Cox models. P < 0.05 (two-sided) was considered to be statistically significant in all analyses.

## Results

### Basic clinic characteristics

Totally 311 patients were enrolled in our study, and basic clinical characteristics were shown in Table 1. There were 214 cases (68.8%) with ST-segment elevation myocardial infarction (STEMI), and 97 cases (31.2%) with non-ST segment elevation myocardial infarction (NSTEMI). CIN occurred in 80 patients (25.7%). Patients in CIN group were older (mean age 71.6 ± 13.0 years vs. 63.8 ± 11.7 years, P <0.001). Compared with non-CIN group patients, CIN group patients had a higher level of GDF-15 and baseline serum creatinine concentration (1232 ± 366.6 ng/L vs. 939.2 ± 309.6 ng/L, 108.2 ± 34.1 ng/L vs. 72.6 ± 16.6 ng/L, all P <0.001) and a lower level of eGFR (56.6 ± 24.0 ml/min/1.73m^2^ vs 88.7 ± 22.3mL/min/1.73m^2^, P <0.001).

**Table 1:** Basic clinical characteristics of CIN group and non-CIN group.

Variables	CIN Group(n = 80)	Non-CIN Group(n = 231)	P-value
**Demographics**			
Age, y	71.6±13.0	63.8±11.7	<0.001
Male, n%	54(67.5%)	166(71.9%)	0.478
BMI, Kg/m^2^	23.4±4.8	23.4±4.5	0.894
SBP, mmHg	123.1±20.3	124.1±19.7	0.954
DBP, mmHg	82.5±21.2	84.4±21.0	0.749
Heart rate, bpm	86.7±15.1	83.9±14.5	0.212
STEMI	59(73.8%)	155(67.1%)	0.327
**Medical history, n%**			
Smoking	37(46.3%)	114(49.4%)	0.697
Drinking,	11(13.8%)	51(22.1%)	0.143
Hypertension	56(70%)	135(58.4%)	0.083
Diabetes	17(21.2%)	54(23.3%)	0.759
**Medications, n%**			
ACEI/ARB	67(83.8%)	196(84.8%)	0.858
β-blocker	74(92.5%)	204(88.3%)	0.4
CCB	7(8.8%)	28(12.1%)	0.539
Diuretics	8(10%)	33(14.3%)	0.443
Statin	77(96.2%)	225(97.4%)	0.699
**Laboratory measurements**			
Serum creatinine, μmol/L	108.2±34.1	72.6±16.6	<0.001
eGFR, ml/min/1.73m^2^	56.6±24.0	88.7±22.3	<0.001
TC,mmol/L	4.3±1.0	4.3±1.1	0.847
HDL-C, mmol/L	1.2±0.3	1.1±0.4	0.064
LDL-C, mmol/L	2.6±0.7	2.6±0.9	0.89
Uric acid, μmol/L	392.2±147.3	379.6±157.6	0.501
Serum albumin, g/L	34.1±3.5	34.2±3.4	0.62
WBC, 10^9^/L	8.7±3.0	8.9±3.3	0.881
Anemia, n%	29(36.3%)	18(7.8%)	<0.001
TSH, μIU/ml	1.38±0.94	1.42±1.08	0.364
HbA1c, %	6.42±1.24	6.37±1.35	0.498
GDF-15, ng/L	1232±366.6	939.2±309.6	<0.001
**Procedural characteristic**			
Contrast volume, mL	174.6±44.7	176.5±44.2	0.75
Contrast exposure time, min	62.9±19.2	60.4±19.6	0.306
Number of stents, n	1.6±0.6	1.5±0.5	0.15
Use of isotonic contrast agents	3(3.8%)	1(0.4%)	0.054
Hydration therapy	11(13.8%)	11(4.8%)	0.011
Primary PCI	59(73.8%)	108(46.8%)	<0.001

CIN = contrast-induced nephropathy, BMI = body mass index, SBP = systolic blood pressure, DBP = diastolic blood pressure, STEMI = ST-segment elevation myocardial infarction, ACEI/ARB = angiotensin-converting enzyme inhibitor/angiotensin receptor blocker, CCB = Calcium channel blocker, eGFR = estimated glomerular filtration rate (mL/min/1.73m^2^), TC = total cholesterol, HDL-C = High-density lipoprotein cholesterol, LDL-C = Low-density lipoprotein cholesterol, WBC = white blood cell, Anemia was defined using World Health Organization criteria: baseline hematocrit value <39% for men and <36% for women, TSH = thyrotropin, thyroid stimulating hormone, GDF15 = growth differentiation factor-15, HbA1c = glycated hemoglobin, PCI = percutaneous coronary intervention.

### Role of GDF-15 in predicting CIN

**Fig 1:**
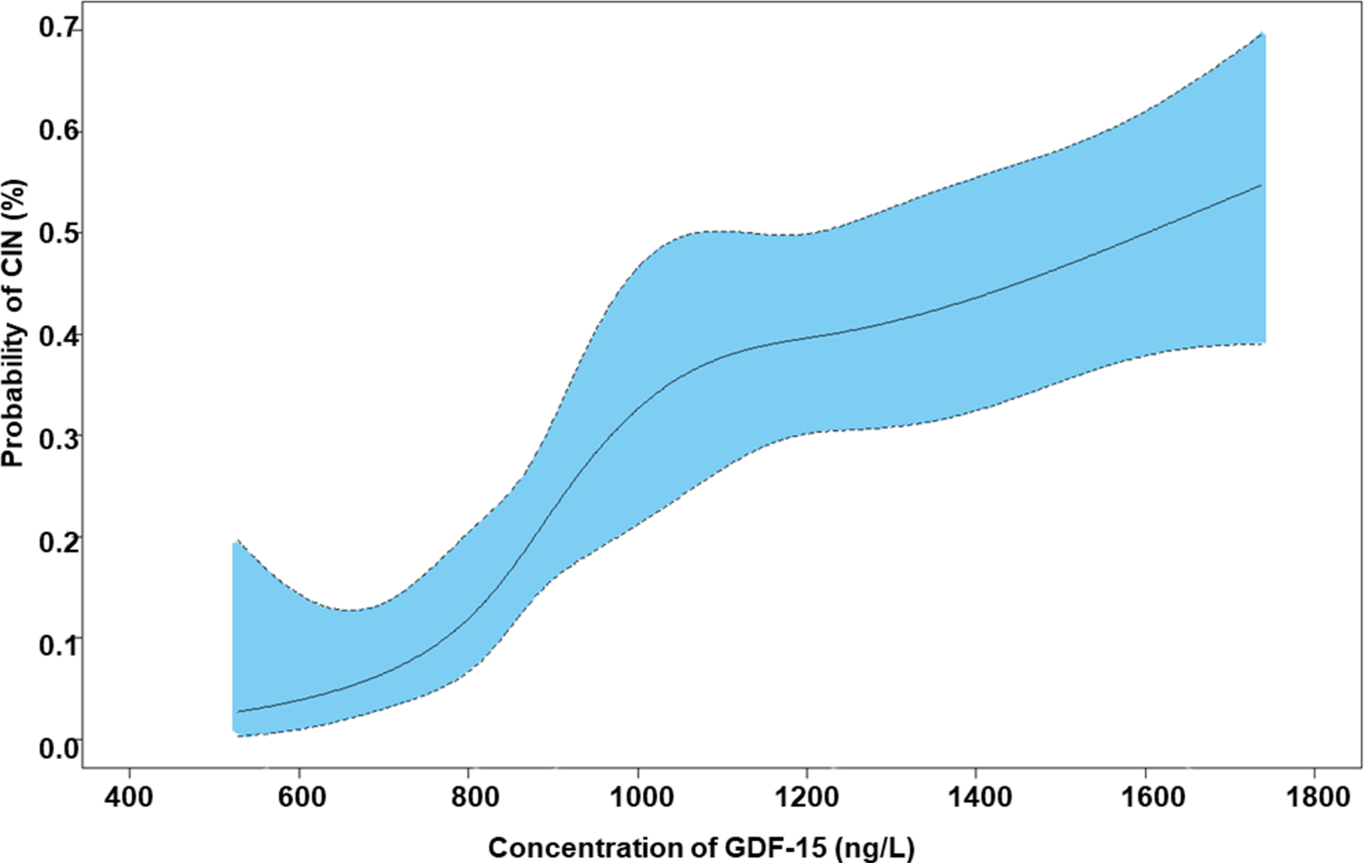
Restricted cubic spline curve of GDF-15 and CIN. X-axis represented the concentration of GDF-15, the blue area showed the 95%CI of GDF-15. Y-axis represented the risk of CIN in our study cohort.

**Fig 2:**
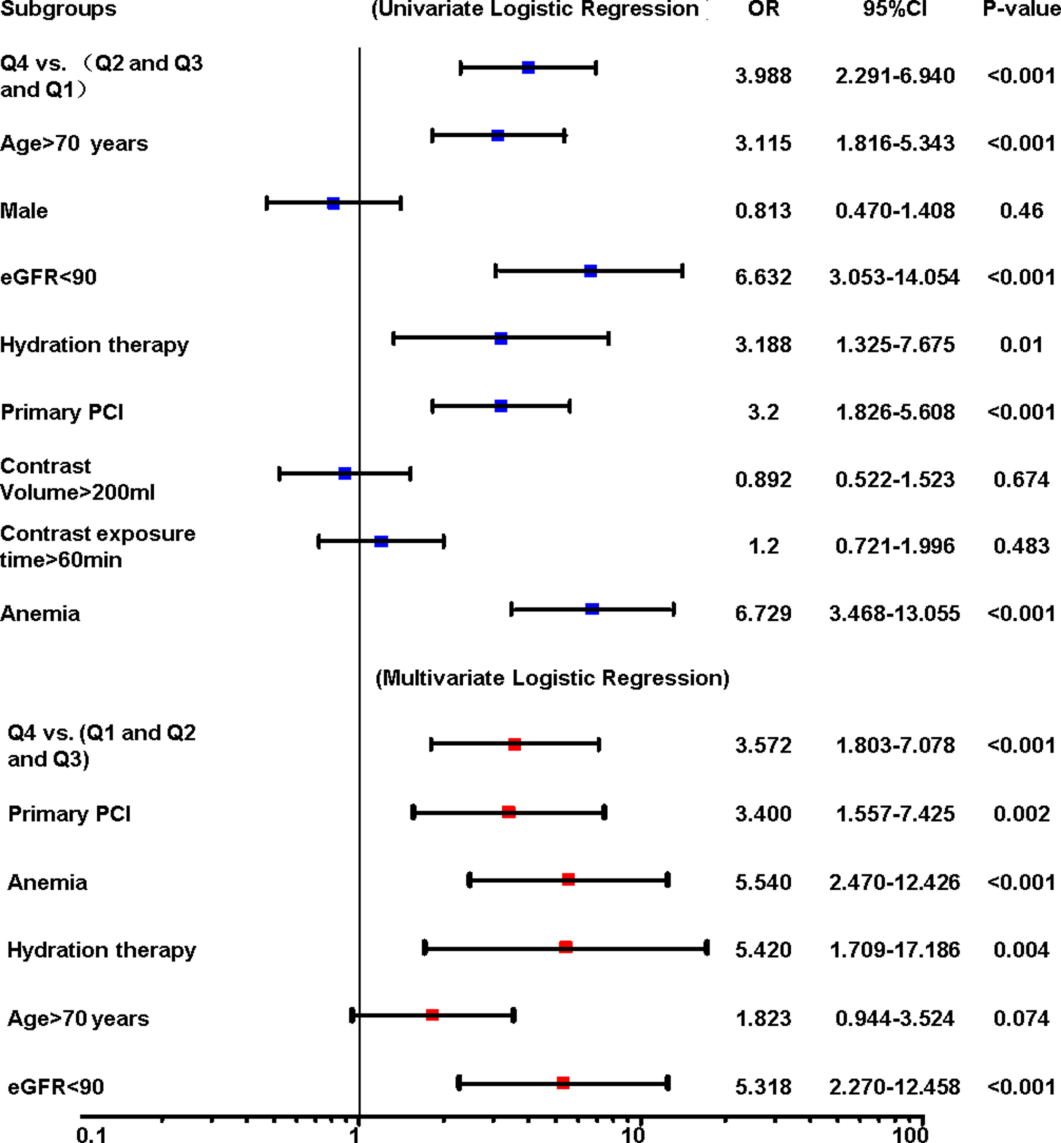
Logistic analysis of independent predictors for CIN. Presented are univariate and multivariate logistic regression analysis to identify independent risk factors for CIN in AMI patients.

According to GDF-15 quartiles, patients were divided into four groups and the baseline characteristics were showed in Table 2. There were significant differences in age, serum creatinine concentration, eGFR, anemia between groups. The incidence of CIN increased according to GDF-15 quartiles. To investigate the association between GDF-15 and CIN, restricted cubic spline curve was showed in Fig 1. The level of GDF-15 and risk of CIN showed positive relevant. As showed in Fig 2, multivariate logistic model indicated that the highest quartiles (Q4) was significantly associated with an increased risk of CIN compared with lower level of GDF-15 (Q1, Q2 and Q3) (OR : 3.572, 95%CI: 1.803–7.078, P <0.001). The independent predictors also included anemia (OR: 5.540, 95%CI: 2.470–12.426, P <0.001), hydration therapy (OR: 5.420, 95%CI: 1.709–17.186, P = 0.004), primary PCI (OR: 3.400, 95%CI: 1.557–7.425, P = 0.002) and eGFR <90mL/min/1.73m^2^ (OR: 5.318, 95%CI: 2.270–12.458, P <0.001).

**Table 2:** Baseline patient characteristics according to GDF-15 quartiles.

Characteristics	GDF-15 Quartiles				
	Q1(GDF-15 ≤800.27 ng/L), n = 78	Q2(800.27ng/L< GDF-15 ≤900.49 ng/L, n = 78)	Q3(900.49ng/L< GDF-15 <1258.98 ng/L, n = 77)	Q4(GDF-15 ≥1258.98 ng/L, n = 78)	P-value
**Demographics**					
Age, y	65.9±10.7	62.6±12.3	66.7±13.6	68.2±12.9	0.039
Male, n%	58(74.4%)	55(70.5%)	52(67.5%)	55(70.5%)	0.830
BMI, Kg/m^2^	23.4±5.1	23.0±4.5	23.6±4.3	23.7±4.4	0.820
SBP, mmHg	120.7±18.9	127.0±19.7	125.5±19.7	122.0±20.7	0.160
DBP, mmHg	80.6±19.7	88.3±21.2	84.3±20.9	82.4±22.1	0.128
Heart rate, bpm	85.3±14.2	84.6±16.3	86.8±14.9	81.8±13.2	0.198
**Medical history, n%**					
Smoking	41(52.6%)	43(55.1%)	31(40.3%)	36(46.2%)	0.245
Drinking,	15(19.2%)	13(16.7%)	16(20.8%)	18(23.1%)	0.786
Hypertension	50(64.1%)	45(57.7%)	47(61.0%)	49(62.8%)	0.858
Diabetes	16(20.5%)	17(21.8%)	18(23.4%)	20(25.6%)	0.885
**Medications, n%**					
ACEI/ARB	65(83.3%)	67(85.9%)	65(84.4%)	66(84.6%)	0.978
β-blocker	71(91.0%)	67(85.9%)	68(88.3%)	72(92.3%)	0.569
CCB	6(7.7%)	13(16.7%)	9(11.7%)	7(9.0%)	0.276
Diuretics	10(12.8%)	10(12.8%)	11(14.3%)	10(12.8%)	0.991
Statin	78(100%)	75(96.2%)	72(93.5%)	77(98.7%)	0.077
**Laboratory measurements**					
Serum creatinine, μmol/L	75.6±15.9	73.7±20.3	88.7±35.4	89.0±30.0	<0.001
eGFR, ml/min/1.73m2	83.7±21.3	89.6±28.8	75.0±28.7	73.2±27.1	<0.001
TC,mmol/L	4.3±1.0	4.2±1.0	4.3±1.0	4.5±1.3	0.470
HDL-C, mmol/L	1.1±0.5	1.1±0.3	1.1±0.4	1.1±0.3	0.830
LDL-C, mmol/L	2.5±0.8	2.5±0.8	2.5±0.7	2.8±1.0	0.124
Uric acid,μmol/L	373.5±150.2	370.8±152.8	396.4±160.1	390.8±157.8	0.673
Serum albumin, g/L	34.6±3.1	33.5±3.4	34.1±3.6	34.5±3.5	0.243
WBC, 10^9^/L	9.3±3.3	8.9±3.4	9.0±2.9	8.2±3.2	0.171
Anemia, n%	3(3.8%)	8(10.3%)	20(26.0%)	16(20.5%)	<0.001
TSH, μIU/ml	1.37±1.25	1.45±1.12	1.34±0.83	1.49±0.96	0.789
HbA1c, %	6.48±1.56	6.22±0.94	6.56±1.48	6.27±1.21	0.339
**Procedural characteristic**					
Contrast volume, mL	178.5±44.4	174.0±44.2	175.2±46.9	176.2±44.2	0.934
Contrast exposure time, min	58.5±21.4	62.9±17.6	61.6±19.6	61.2±19.4	0.550
Number of stents	1.4±0.5	1.6±0.5	1.5±0.6	1.5±0.5	0.404
Use of isotonic contrast agents	0(0.0%)	0(0.0%)	3(3.9%)	1(1.3%)	0.104
Hydration therapy	5(6.4%)	3(3.8%)	7(9.1%)	7(9.0%)	0.533
CIN	3(3.8%)	15(19.2%)	25(32.5%)	37(47.4%)	<0.001
Primary PCI	35(44.9%)	37(47.4%)	43(55.8%)	52(66.7%)	0.028

Presented are clinical characteristics of patients according to GDF-15 quartiles

**Fig 3:**
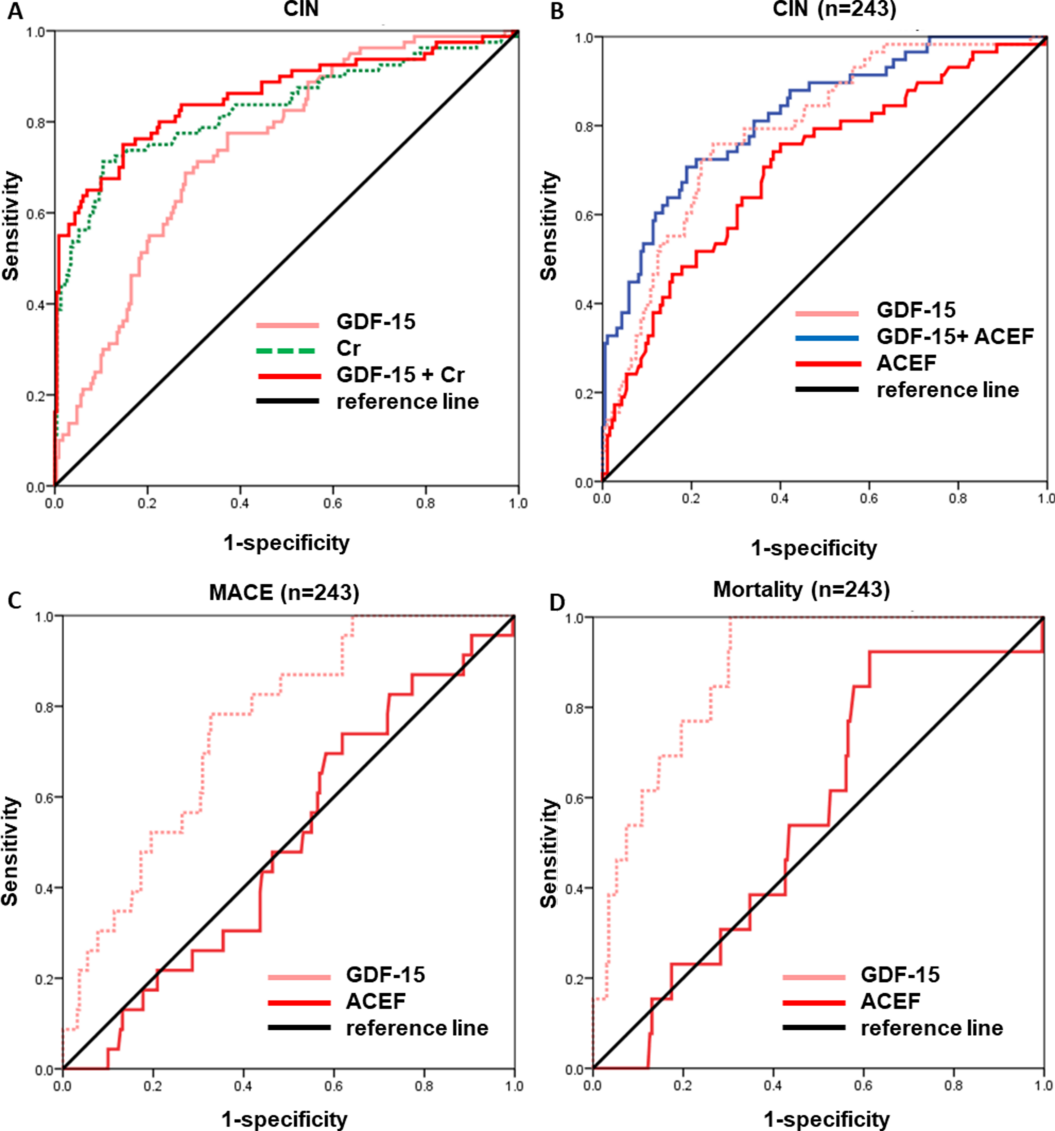
Receiver operator characteristic (ROC) curves of GDF-15, serum creatinine and ACEF risk score. A) Area under the curve (AUC) of GDF-15 was 0.744 (95%CI: 0.685–0.803, P <0.001), AUC of serum creatinine (Cr) was 0.830 (95%CI: 0.770–0.891, P<0.001), and the combination of GDF-15 with serum creatinine showed a better performance than the two biomarker alone (AUC: 0.859, 95%CI: 0.794–0.925, P < 0.001). It indicated that GDF-15 was of certain value to predict CIN, serum creatinine had a better performance in predicting CIN than GDF-15 (P = 0.030). However, incorporating GDF-15 to serum creatinine could not provide a better predictive value than serum creatinine alone (P = 0.048). B) Of the 243 patients, the predictive values of GDF-15 and the ACEF risk score were compared. AUC of GDF-15 was 0.793, 95%CI: 0.729–0.856, P < 0.001 (n = 243), while AUC of ACEF was 0.708, 95%CI: 0.630–0.786, P < 0.001. The combination of GDF-15 and the ACEF risk score showed an AUC of 0.824, 95%CI: 0.763–0.885, P < 0.001. The efficiency in predicting CIN is the same by GDF-15 or ACEF alone (P = 0.099), while model with both GDF-15 and ACEF significantly enhance the predicting efficiency than ACEF alone (P = 0.001). C) It showed an AUC of 0.795 (95%CI: 0.665–0.843, P < 0.001) for GDF-15 to predict MACE event. D) It showed an AUC of 0.881 (95%CI: 0.665–0.843, P < 0.001) for GDF-15 to predict mortality.

As showed in Table 3, ROC analysis revealed that the AUC of GDF-15 was 0.744, 95%CI: 0.685–0.803, P < 0.001, whereas the AUC of serum creatinine was 0.830, 0.770–0.891, P < 0.001. It indicated that there were of certain value for GDF-15 to predict CIN. Serum creatinine had a better performance in predicting CIN than GDF-15 (P = 0.030). However, adding GDF-15 to serum creatinine or eGFR could provide a better predictive value than serum creatinine or eGFR alone (Fig 3A). There were also of certain values for other variables from logistic model except hydration therapy.

**Table 3:** AUC of variables for predicting CIN.

Variables	AUC	95%CI	P value
GDF-15	0.744	0.685–0.803	<0.001
Serum creatinine^a^	0.830	0.770–0.891	<0.001
eGFR	0.853	0.796–0.910	<0.001
Age	0.686	0.614–0.757	<0.001
Hydration therapy	0.545	0.469–0.621	0.231
Primary PCI	0.635	0.566–0.704	<0.001
Anemia	0.642	0.566–0.718	<0.001
GDF-15 plus serum creatinine^b^	0.857	0.802–0.912	<0.001
GDF-15 plus eGFR^b^	0.873	0.820–0.926	<0.001

Presented are AUC of GDF-15 and other variables for predicting CIN.

^a^ Serum creatinine had a better performance in predicting CIN than GDF-15 (P = 0.030).

^b^ Adding GDF-15 to serum creatinine or eGFR could provide a better predictive value than serum creatinine or eGFR alone (P = 0.048; P<0.001).

### Performance of GDF-15 versus. ACEF risk score

The predictive value of GDF-15 and ACEF risk score were compared in 243 patients who determined left ventricular ejection fraction (LVEF) in our study. AUC of GDF-15 was 0.793, 95%CI: 0.729–0.856, P <0.001, while AUC of ACEF was 0.708, 95%CI: 0.630–0.786, P <0.001 (Fig 3B). These results showed same accuracy in predicting CIN by GDF-15 or ACEF alone (P = 0.099), while GDF-15 with ACEF significantly enhance the predicting efficiency than ACEF alone (P = 0.001).

**Fig 4:**
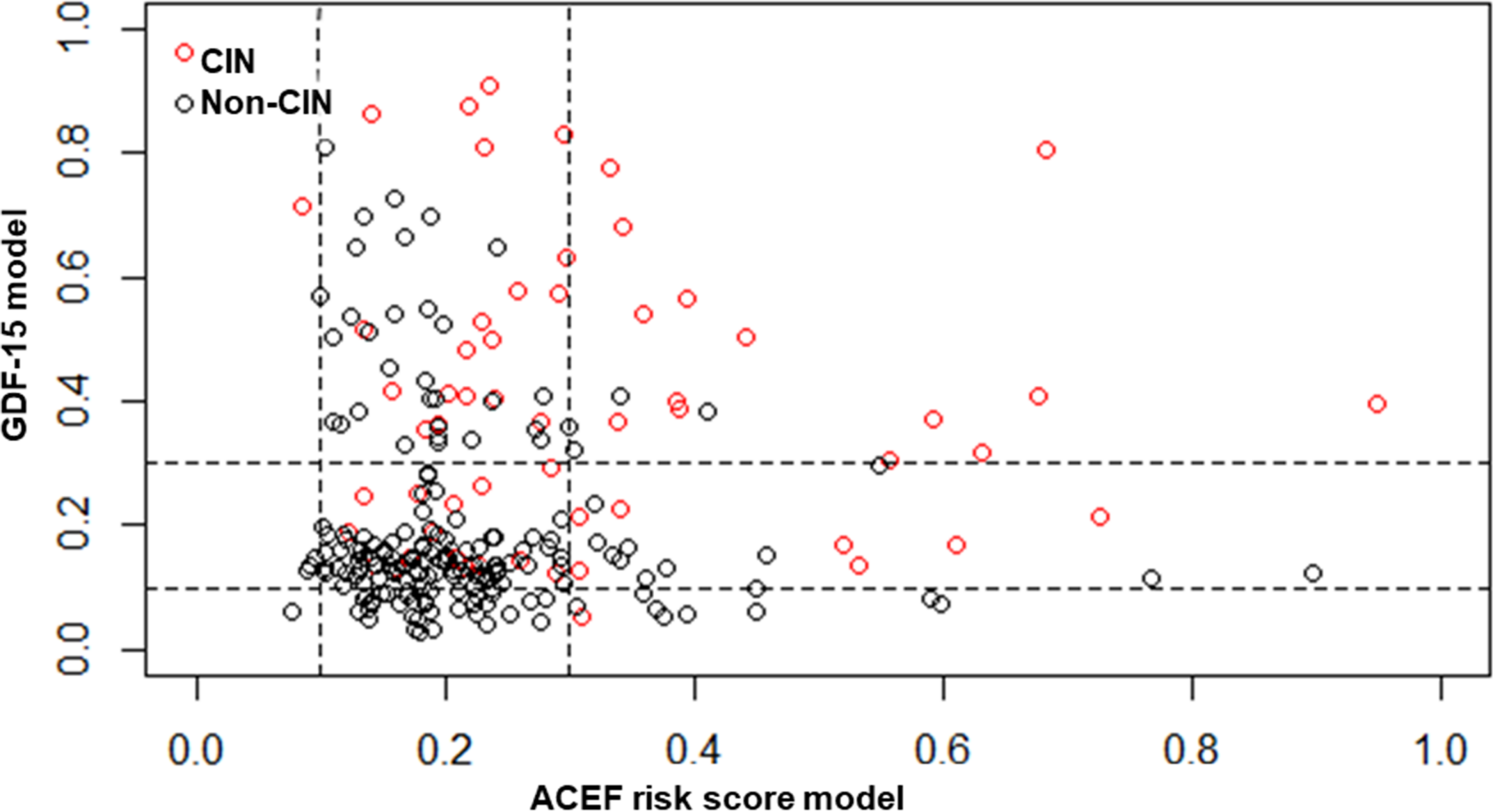
The distribution of all enrolled patients in GDF-15 model and the ACEF risk model. Using 10% and 30% as arbitrary thresholds to define patients at low, intermediate, and high risk, we could intuitive take a look at the distribution of our study population. X-axis represents the ACEF risk score, while y-axis represents the GDF-15 model. The red circle represents the CIN group patients and the black circle represents the non-CIN group patients.

NRI was calculated to compare potential clinical benefits achieved by GDF-15 model with ACEF risk score. Using 10% and 30% as arbitrary thresholds to define patients at low, intermediate, and high risk, GDF-15 achieved an NRI of 0.32 (95%CI: 0.123–0.518, P = 0.001) compared with ACEF risk score. Of 185 patients without events, 55 were correctly downgraded and 34 were wrongly upgraded by at least one category by GDF-15 (NRI for non-events = 0.114), whereas of 58 patients with events, 20 were correctly upgraded, and 8 was wrongly downgraded (NRI for events = 0.207) (Table 4). The distribution of each case in two models was showed in Fig 4.

**Table 4:** Reclassification across pre-defined risk thresholds in a cohort of 243 patients using GDF-15 and the ACEF risk score.

Patients with events				
	GDF-15 model			
the ACEF risk model	<10%	10%-30%	>30%	ALL
<10%	0	0	1	1
10%-30%	0	16	19	35
>30%	1	7	14	22
ALL	1	23	34	58
				NRI = 0.207
Patients without events				
	GDF-15 model			
the ACEF risk model	<10%	10%-30%	>30%	ALL
<10%	1	3	1	5
10%-30%	35	92	30	157
>30%	9	11	3	23
ALL	45	106	34	185
				NRI = 0.114

Presented are the number of patients in each risk category. Patients were divided into subgroups that did or did not reach the endpoint of CIN. NRI = net reclassification improvement. Total category-based NRI was 0.32 (95% CI: 0.123–0.518, P = 0.001)

### Outcome data

**Fig 5:**
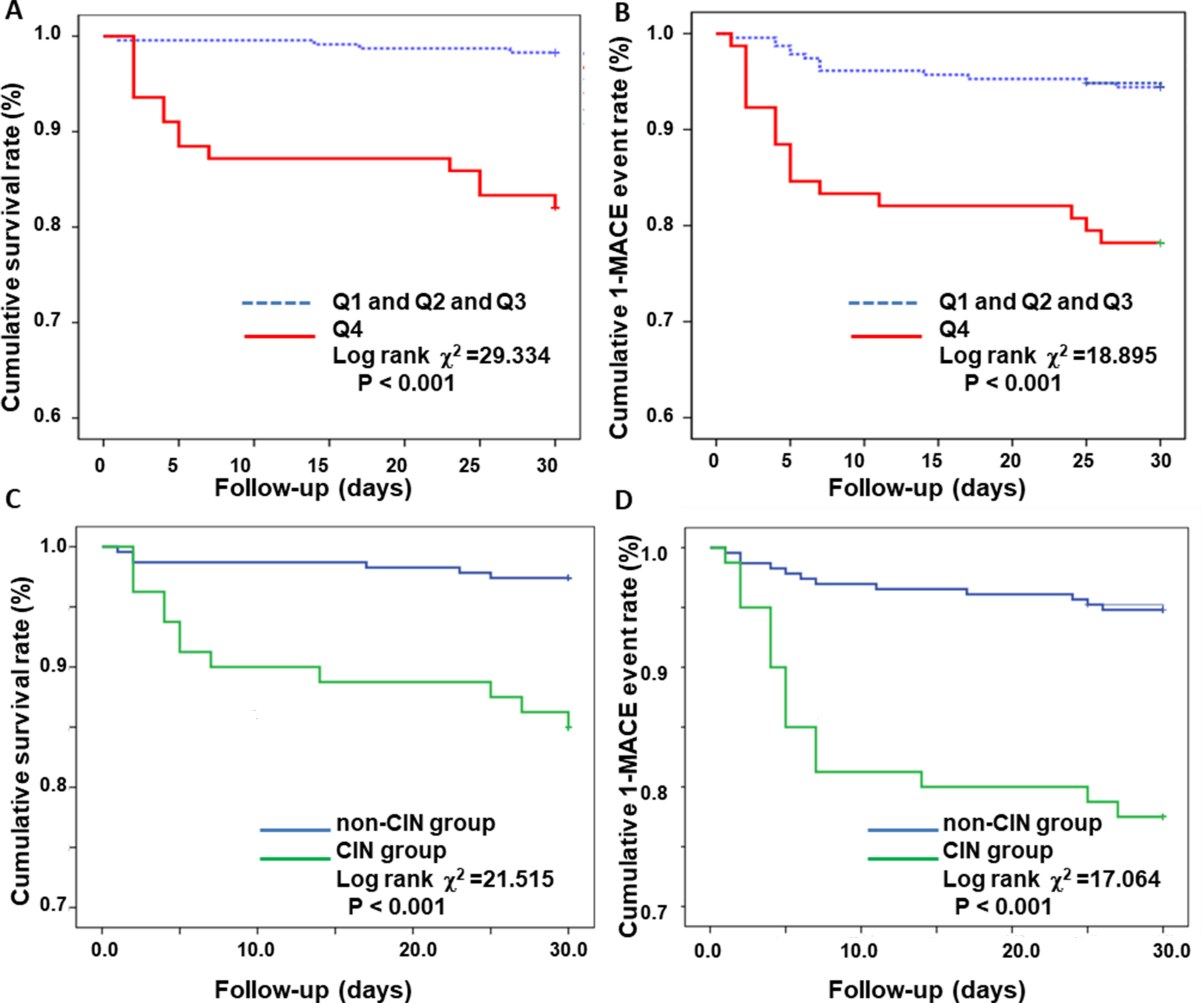
Kaplan-Meier survival curves according to GDF-15 quartiles and the prevalence of CIN. Q1 (GDF-15 ≤ 800.27 ng/L), n = 78, Q2 (800.27ng/L< GDF-15 ≤900.49 ng/L), n = 78, Q3 (900.49ng/L <GDF-15 <1258.98 ng/L), n = 77, Q4 (GDF-15 ≥1258.98 ng/L), n = 78. Presented are Kaplan-Meier survival curves in AMI patients between different GDF-15 quartiles. 30-days survival rate according to GDF-15 quartiles (Q4 vs. Q1 and Q2 and. Q3) (Fig 5A). 30-days 1-MACE event rate in GDF-15 quartiles (Fig 3B). 30-days survival rate between CIN group and non-CIN group (Fig 5C). 30-days 1-MACE event rate between CIN group and non-CIN group (Fig 5D).

Follow-up at an average time of 28 days was carried out to observe the short-term outcome in AMI patients. Based on Kaplan-Meier analysis, the risk of all-cause mortality and MACE during follow-up increased in highest GDF-15 quartile (Q4 vs. Q1 and Q2 and Q3) (both P < 0.001; Fig 5A and 5B). It also indicated a significantly worse prognosis in CIN group compared with non-CIN group (both P < 0.001; Fig 5C and 5D).

**Fig 6:**
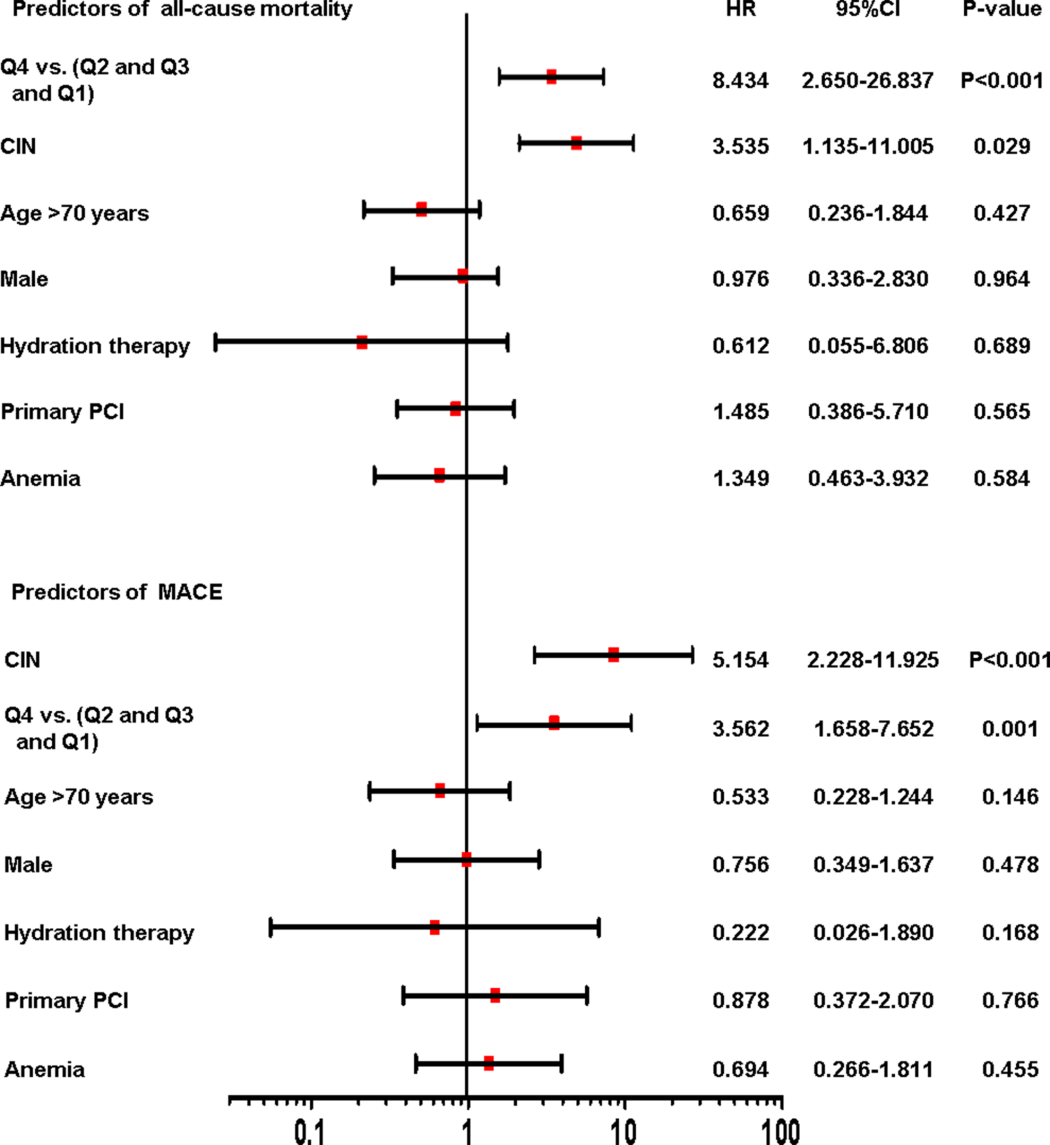
Multivariate Cox analysis: Independent predictors of all-cause mortality and MACE event. Presented are Cox proportional hazard model to estimate the associations between risk factors and short-term outcomes.

**Fig 7:**
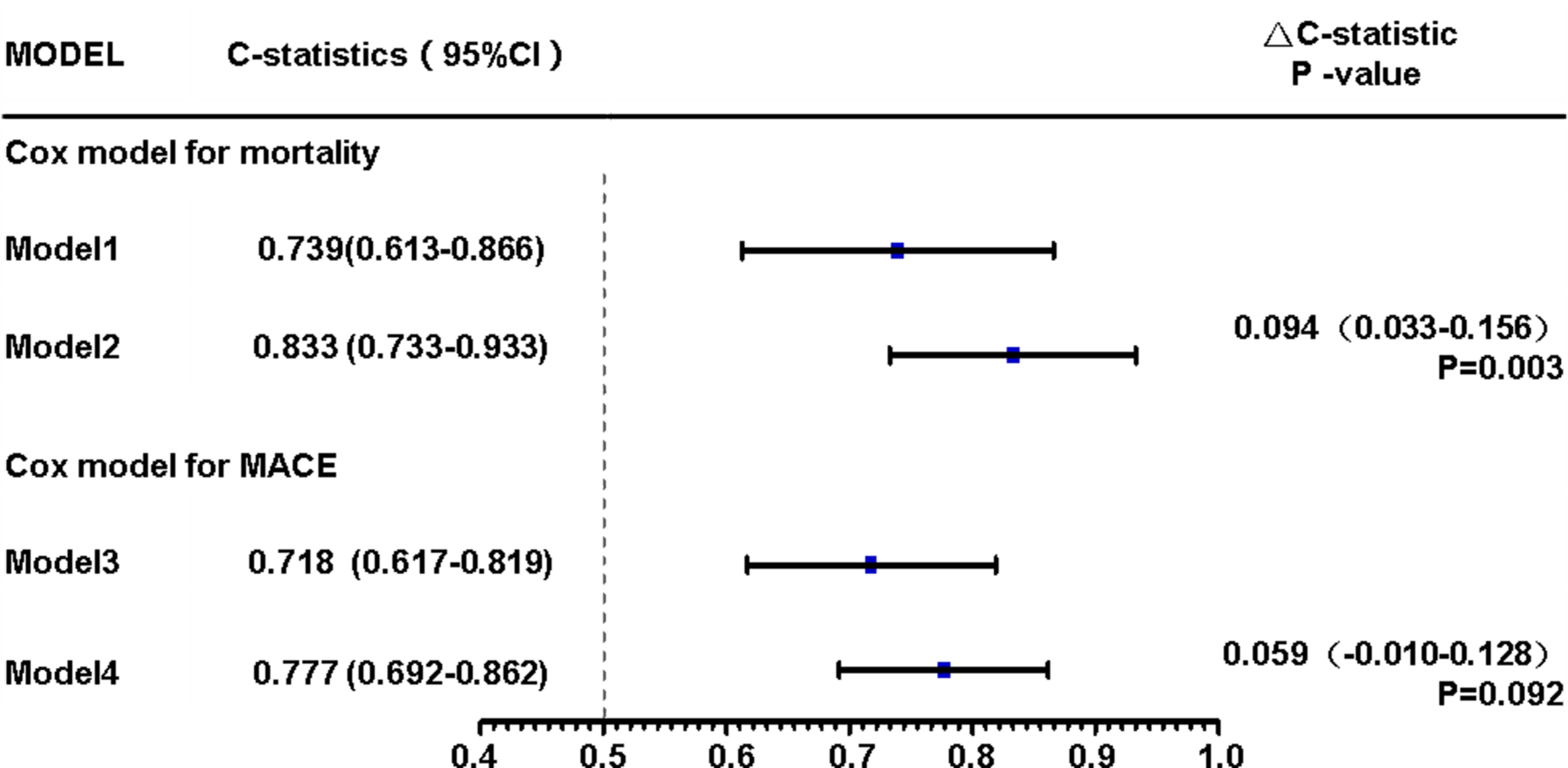
C-statistics of Cox models with or without GDF-15. There were incremental trends in C-statistics incorporating GDF-15 in Cox model. Model 1 (Cox model predicting mortality in Fig 6, but without GDF-15): CIN, Age>70 years, Male, Hydration therapy, Primary PCI, Anemia; Model 2 (Cox model for mortality in Fig 6): Q4 vs.(Q1+Q2+Q3), CIN, Age>70 years, Male, Hydration therapy, Primary PCI, Anemia; Model 3 (Cox model for MACE in Fig 6, but without GDF-15): CIN, Age>70 years, Male, Hydration therapy, Primary PCI, Anemia; Model 4 (Cox model for MACE in Fig 6): Q4 vs.(Q1+Q2+Q3), CIN, Age>70 years, Male, Hydration therapy, Primary PCI, Anemia.

As showed in Fig 6, a Cox regression model including CIN as a variable showed that after adjusting for age, gender, hydration therapy, primary PCI and anemia, the higher level of GDF-15 (Q4) was significantly associated with an increased risk of all-cause mortality and MACE compared with lower GDF-15 quartiles (Q1, Q2 and Q3) (HR: 8.434, 95% CI: 2.650–26.837, P <0.001; HR: 3.562, 95% CI: 1.658–7.652, P = 0.001). It also revealed that CIN is an independent predictor of all-cause mortality and MACE (HR: 3.535, 95% CI: 1.135–11.005, P = 0.029; HR: 5.154, 95% CI: 2.228–11.925, P < 0.001). C-statistics of the Cox model with or without GDF-15 were listed in Fig 7. There were incremental trends in C-statistic with GDF-15 in the model to predict mortality (P = 0.003). However, there were no significant differences when GDF-15 was added in the Cox model to predict MACE (P = 0.092). ROC analysis showed that GDF-15 had a better performance in predicting all-cause mortality than serum creatinine or eGFR (Table 5). However, models incorporating serum creatinine or eGFR with GDF-15 did not provide a better performance than GDF-15 alone in predicting mortality.

**Table 5:** AUC of variables for predicting mortality.

Variables	AUC	95%CI	P value
GDF-15	0.862	0.804–0.921	P<0.001
Serum creatinine	0.706	0.575–0.836	0.003
eGFR	0.710	0.578–0.841	0.003
GDF-15 plus serum creatinine^a^	0.859	0.794–0.925	<0.001
GDF-15 plus eGFR^a^	0.862	0.790–0.933	<0.001

Presented are AUC of GDF-15 and other biomarkers for predicting mortality.

^a^ Adding serum creatinine or eGFR to GDF-15 couldn’t provide a better performance than GDF-15 alone (both P>0.05).

## Discussion

Our study showed that: a) The GDF-15 levels increased in CIN group in AMI patients underwent PCI, GDF-15 was an independent risk factor of CIN in patients with AMI underwent PCI; b) The combination of GDF-15 and serum creatinine provided a better predictive value for CIN, ACEF risk score incorporating GDF-15 showed a better performance in predicting CIN; c) Increased GDF-15 levels and CIN are independent risk factors for all-cause mortality and MACE event in short-term follow-ups.

CIN is the leading cause of acute renal failure in patients with cardiac diseases, leading to high all-cause mortality and adverse consequences after coronary intervention[14] and other clinical practice such as computed tomography scans, angiographies [15]. CIN was reported to be > 2% in the general population and 30% in the population with high risk (elderly, eGFR <60mL/min/1.73m^2^, congestive heart failure, and diabetes mellitus)[3], (varied from 5–30%) [16, 17]. In our study, the incidence rate of CIN in patients undergoing PCI was about 20%. We assume that this may be because our patients enrolled are of advanced age and impaired baseline renal function. Moreover, the definition of CIN differed in different studies. In our study, CIN was defined as an increase of >25% or the absolute increase of >0.5 mg/dL by baseline serum creatinine within 48 or 72 hours. The different time we re-test the serum creatinine concentration might influence the incidence of CIN.

The pathophysiology of CIN is still uncertain. Cellular toxicity, endothelial dysfunction, and tubular apoptosis from hypoxia damage or reactive oxygen species might be the result of CIN [18]. It is important to identify high-risk subjects in the early stages to prevent CIN. In the previous study, the incidence of postoperative acute kidney injury increased in patients with high level of preoperative plasma GDF-15, indicating that GDF-15 is useful for risk stratification of CIN in patients with normal creatinine[19, 20]. Our study further explored the associations between GDF-15 level and CIN in AMI patients underwent PCI. Our results also showed that the incidence of CIN increased in higher GDF-15 level group. GDF-15 is of certain value of in predicting CIN. The combination of GDF-15 and serum creatinine showed a better predictive value than two variables alone. Our results also showed ACEF risk score incorporating GDF-15 provided a better predicting value for CIN.

GDF-15 is a member of transforming growth factor-β superfamily and is increasingly expressed in adipocytes, cardiomyocytes, macrophages, endothelial cells, and vascular smooth muscle cells in experimental conditions[21, 22]. In human hearts, expression of GDF-15 increased during ischemia or reperfusion, which protect the heart from injury[23]. GDF-15 was also reported to be a reliable biomarker to predict the short-term [24]and long-term outcomes of patients with AMI [25–28]. High levels of GDF-15 are associated with increased risks of outcomes in patients with coronary no-reflow [29], acute myocardial infarction[8] and renal dysfunction[30]. Our results also showed GDF-15, serum creatinine and eGFR are effective to predict mortality. We also found that risk of all-cause mortality and MACE during follow-up increased in patients with higher GDF-15 level, which was consistent with previous studies.

Except for GDF-15, Our logistic regression model showed that anemia, primary PCI and eGFR <90ml/min/1.73m^2^ are also independent risk factors for CIN, which is coinciding with previous study[31–33]. Roman reported that anemia and renal dysfunction were predictors of CIN in patients undergoing primary PCI[34]. Other predictors for CIN include old age, hypertension, diabetes mellitus, high contrast dose, multi-vessel diseases, et al[31–33]. In our study, we didn’t find their predictive value of these predictors. This may be because of the differences of the study cohort and sample size. Hydration therapy is an effective method to prevent contrast nephropathy. However, our logistic regression showed that the hydration treatment is a risk factor for CIN. We assume that the reasons for this phenomenon was selection bias, in which patients underwent hydration treatment were of worsen kidney function. Moreover, the results indicated that after hydration treatment, eGFR < 90 are still independent risk factors for CIN. It is necessary to explore more effective measures to prevent CIN.

There are several limitations in our present study. Firstly, this study is a retrospective observational study in a single medical center. Secondly, our study included a relatively small sample size, the validation of GDF-15 to predict CIN after PCI is required to verify in more cohorts or centers. Thirdly, we may miss peak serum creatinine concentration values after PCI, and we didn’t measure serum creatinine concentration in follow-ups, which may cover the real incidence of CIN.

In summary, we found that higher GDF-15 level on admission was associated with increased incidence of CIN. GDF-15 was an independent risk factor of CIN in AMI patients underwent PCI. The combination of GDF-15 and serum creatinine was of certain value in predicting CIN. Higher GDF-15 levels and CIN are independent risk factors for all-cause mortality and MACE event in short-term follow-ups.

## Supporting information

S1 Table Minimal data set. HBP = high blood pressure, SBP = systolic blood pressure, DBP = diastolic blood pressure, HR = heart rate, WBC = white blood cell count, CR = creatinine, GDF-15 = growth differentiation factor-15, CIN = contrast-induced nephropathy, MACE = major adverse clinical events, EF = ejection fraction.
(XLSX)
